# Identification and validation of DOCK4 as a potential biomarker for risk of bone metastasis development in patients with early breast cancer

**DOI:** 10.1002/path.5197

**Published:** 2019-01-25

**Authors:** Jules A Westbrook, Steven L Wood, David A Cairns, Kathryn McMahon, Renu Gahlaut, Helene Thygesen, Mike Shires, Stephanie Roberts, Helen Marshall, Maria R Oliva, Mark J Dunning, Andrew M Hanby, Peter J Selby, Valerie Speirs, Georgia Mavria, Robert E Coleman, Janet E Brown

**Affiliations:** ^1^ Department of Oncology and Metabolism, Academic Unit of Clinical Oncology University of Sheffield Sheffield UK; ^2^ Clinical and Biomedical Proteomics Group Leeds Institute of Cancer and Pathology, University of Leeds Leeds UK; ^3^ Clinical Trials Research Unit, Leeds Institute of Clinical Trials Research University of Leeds Leeds UK; ^4^ Leeds Institute of Cancer and Pathology University of Leeds Leeds UK; ^5^ Sheffield Institute of Translational Neuroscience University of Sheffield Sheffield UK; ^6^ Institute of Medical Sciences University of Aberdeen Aberdeen UK

**Keywords:** DOCK4, bone metastasis, breast cancer, biomarker, proteomics

## Abstract

Skeletal metastasis occurs in around 75% of advanced breast cancers, with the disease incurable once cancer cells disseminate to bone, but there remains an unmet need for biomarkers to identify patients at high risk of bone recurrence. This study aimed to identify such a biomarker and to assess its utility in predicting response to adjuvant zoledronic acid (zoledronate). We used quantitative proteomics (stable isotope labelling by amino acids in cell culture‐mass spectrometry; SILAC‐MS) to compare protein expression in a bone‐homing variant (BM1) of the human breast cancer cell line MDA‐MB‐231 with parental non‐bone‐homing cells to identify novel biomarkers for risk of subsequent bone metastasis in early breast cancer. SILAC‐MS showed that dedicator of cytokinesis protein 4 (DOCK4) was upregulated in bone‐homing BM1 cells, confirmed by western blotting. BM1 cells also had enhanced invasive ability compared with parental cells, which could be reduced by DOCK4‐shRNA. In a training tissue microarray (TMA) comprising 345 patients with early breast cancer, immunohistochemistry followed by Cox regression revealed that high DOCK4 expression correlated with histological grade (*p* = 0.004) but not oestrogen receptor status (*p* = 0.19) or lymph node involvement (*p* = 0.15). A clinical validation TMA used tissue samples and the clinical database from the large AZURE adjuvant study (*n* = 689). Adjusted Cox regression analyses showed that high DOCK4 expression in the control arm (no zoledronate) was significantly prognostic for first recurrence in bone (HR 2.13, 95%CI 1.06–4.30, *p* = 0.034). No corresponding association was found in patients who received zoledronate (HR 0.812, 95%CI 0.176–3.76, *p* = 0.790), suggesting that treatment with zoledronate may counteract the higher risk for bone relapse from high DOCK4‐expressing tumours. High DOCK4 expression was not associated with metastasis to non‐skeletal sites when these were assessed collectively. In conclusion, high DOCK4 in early breast cancer is significantly associated with aggressive disease and with future bone metastasis and is a potentially useful biomarker for subsequent bone metastasis risk. Copyright © 2018 Pathological Society of Great Britain and Ireland. Published by John Wiley & Sons, Ltd.

## Introduction

Despite substantial progress in early detection and treatment, breast cancer still accounts for 15% of female cancer‐related deaths, with skeletal metastasis occurring in over 70% of patients with advanced disease 1. Relapse in bone typically occurs years after apparently successful treatment of early breast cancer and a period of tumour dormancy. Bone‐targeted agents, such as bisphosphonates 2 and denosumab 3, are widely used to treat the skeletal complications of established bone metastases, but have also recently been the focus of several large adjuvant studies in early breast cancer to assess their potential to reduce the frequency of relapse in bone and subsequent breast cancer mortality. The phase III AZURE trial (BIG01/04‐ISRCTN79831382) recruited 3360 patients with stage II/III breast cancer randomised (1:1) to 5 years of standard adjuvant therapy alone (control) or standard therapy with zoledronic acid (zoledronate) 4. Although there was no significant difference in invasive disease‐free survival (DFS) in the overall population, zoledronate improved disease outcomes for women who were >5 years postmenopausal at diagnosis and a meta‐analysis of 26 randomised trials (*N* = 18 766) demonstrated that bone recurrences (HR = 0.72; 95%CI 0.60, 0.86, 2*p* = 0.0002) and breast cancer deaths (HR 0.82; 95%CI 0.73, 0.93, 2*p* = 0.002) were reduced by adjuvant bisphosphonates in postmenopausal women 5. Breast cancer practice has changed as a result of these studies, but they also highlight the unmet need for biomarkers to identify patients with early breast cancer who are most at risk of developing bone recurrence, thus permitting tailoring of treatment to patients who would probably benefit and sparing patients who would not benefit from potential complications 6.

Proteomic studies are yielding key information about breast cancer metastasis to bone 7, 8, 9 and, in a recent proteomics‐based study, validated in 571 patients, we showed that the proteins CAPG and GIPC1 had both prognostic and predictive potential as biomarkers of bone metastasis 10. In the current study, we hypothesised that proteins, identified by proteomics and upregulated in breast cancer cells that have a propensity to home to bone, would be potential biomarkers for metastasis and could play key mechanistic roles in the process of metastatic dissemination to bone.

## Materials and methods

### Proteomic studies

(See supplementary material, Supplementary materials and methods and Figure S1 for more details).

#### Cell culture and stable isotope labelling by amino acids in cell culture (SILAC)

The human breast cancer cell line MDA‐MB‐231 (parental control cells [PCC] obtained originally from ATCC, Teddington, Middlesex, UK) and a bone‐homing variant (BM1, bone metastatic cells) 11 (supplied by Professor Joan Massagué, Sloan‐Kettering Institute, New York, NY, USA) were used in a ‘classical’ SILAC experiment 12, 13. PCC and BM1 cells were cultured in ‘heavy’ SILAC medium and ‘light’ medium for approximately 10 doublings to ensure >95% isotope label incorporation (heavy media); all cells were used within 10 doublings. ‘Heavy’ SILAC media consisted of DMEM containing l‐arginine (^13^C_6_, ^15^N_4_) and l‐lysine (^13^C_6_, ^15^N_2_) (R10K8, DMEM‐15, Dundee Cell Products, Dundee, UK) supplemented with dialysed (10 kDa molecular weight cut‐off [MWCO]) FBS (D‐FBS100, Dundee Cell Products). ‘Light’ media was DMEM but without the heavy isotopes (R0K0) and with 10% dialysed (10 kDa MWCO) FBS.

#### LC–MS/MS

Equal amounts of protein (40 μg) from light‐ and heavy‐labelled samples were combined, reduced, alkylated and separated on a 1D SDS‐PAGE gel. Liquid‐chromatography tandem‐mass spectrometry (LC–MS/MS) was performed by Dundee Cell Products. Ten slices of the gel‐resolved proteins were cut and proteins were digested into peptides using trypsin. Tryptic peptides were separated using a nanoflow LC‐System coupled to an LTQ‐Orbitrap mass spectrometer (ThermoFisher Scientific, Warrington, UK).

#### Quantitation and bioinformatics analysis

Quantitation was performed using the software Max Quant (http://www.maxquant.org/downloads.htm), with peptide ratios calculated for each arginine‐ and/or lysine‐containing peptide as the peak area of labelled arginine/lysine divided by the peak area of non‐labelled arginine/lysine for each single‐scan mass spectrum. Peptide ratios for all arginine‐ and lysine‐containing peptides sequenced for each protein were averaged. Data output from Max Quant were analysed further using Excel and R (v. 3.2, http://www.r‐project.org/) to select differentially expressed proteins.

### Western blotting

The differential expression of dedicator of cytokinesis protein 4 (DOCK4), and confirmation of DOCK4 knockdown, was assessed in cell lysates using western blotting and an infrared (IR) immunodetection system (LI‐COR Biosciences, Lincoln, NE, USA), as well as by ECL (Promega, Southampton, UK). The primary antibodies used were: DOCK4 (Abcam, Cambridge, UK; ab56743, mouse monoclonal, 0.1 μg/ml); beta‐tubulin (Abcam; ab6046, rabbit polyclonal, 1:5000 dilution). Secondary antibodies were: #925‐68070 IRDye 680RD goat anti‐mouse IgG (H + L), LI‐COR Biosciences, 1:5000 dilution, for the detection of DOCK4 in the 700 nm channel (red); #925‐32211 IRDye 800CW goat anti‐rabbit IgG (H + L), LI‐COR Biosciences, 1:5000 dilution, for the detection of beta‐tubulin in the 800 nm channel (green). For ECL, secondary antibodies were goat anti‐mouse‐HRP (Abcam; ab6789) and goat anti‐rabbit‐HRP (Abcam; ab6721), both 1:2500. Normalised densitometric data from six replicate runs of the fluorescent‐antibody immunoprobed samples (and three blots from the ECL‐visualised samples), were tested for significance using Student's *t*‐test.

### Generation of cell lines with stable DOCK4 knockdown and 3D invasion assay

DOCK4 expression was knocked down in the BM1 and PCC cell lines by lentiviral delivery of a validated shRNA targeting DOCK4, with the use of an empty vector (control vector) as control 14. Wild‐type cells were also studied as a control. Inclusion of a GFP marker protein within the lentiviral vectors enabled Fluorescence activated cell sorting (FACS) separation of vector‐bearing clones and subsequent culture of pure cell populations. Infected cells were selected by FACS sorting 48 h after lentiviral infection. Confirmation of knockdown of DOCK4 protein expression was carried out by western blotting as described above. Quantification of the blots was performed by densitometric scanning.

To study the effects of reduced expression of DOCK4 protein in PCC and BM1, an invasion assay was carried out using the IncuCyte^®^ platform (Essen BioScience, Welwyn Garden City, UK) following the manufacturer's instructions for a ‘scratch‐wound’ procedure. In brief, triplicate wells of an ImageLock 96‐well plate (Essen BioScience 4379) were overlaid with thin Matrigel onto which wild‐type, control vector cells or shRNA‐treated cells of PCC (50 000 cells/well) and BM1 cell lines (100 000 cells/well) were seeded and a homogeneous scratch was created using the Incucyte^®^ WoundMaker tool when the cell monolayer was confluent. This system enables assessment of both cell invasion and migration ability in a single assay. Cell invasion/migration was assessed by measuring the closure of the scratch introduced on the confluent cell monolayer. Experiments were performed in triplicate using independent cultures of cells. Data were analysed using IncuCyte^®^ software and Excel.

### Patients, samples and immunohistochemistry

All analyses on patient samples were performed with ethics approval (Leeds training set, 06/Q1206/180; AZURE validation set 55/03/182).

Initial studies of DOCK4 expression were performed using a training tissue microarray (TMA) comprising 345 specimens with defined tumour type, grade, oestrogen receptor (ER), lymph node and overall survival data, from breast tumours diagnosed at the Leeds Teaching Hospitals NHS Trust (1987–2005). Samples were stained using a rabbit polyclonal DOCK4 antibody (1:100, Bethyl Laboratories Inc., Montgomery, TX, USA; A302‐263A) and corroborated in a smaller cohort with a mouse monoclonal antibody (1:50, Abcam; ab56743). Additional details are provided in supplementary material, Supplementary materials and methods.

The main analyses for correlations with risk of bone metastasis were performed on TMAs constructed from primary tumours from a subset of patients within the overall AZURE trial (*n* = 689). Due to the relatively high prevalence of bone metastatic outcomes and the long follow‐up (median 84 months [interquartile range 66–93]), these TMAs provide an excellent resource for validation of protein biomarkers emerging from our proteomics studies. Protein expression was assessed using the Bethyl DOCK4 antibody (A302‐263A). DOCK4 specificity for this antibody was confirmed by immunohistochemistry using formalin‐fixed paraffin‐embedded (FFPE) cell pellets from BM1 cells with and without DOCK4 knockdown (see supplementary material, Figure S2). Full gel images for the antibodies using ECL as the visualisation method with use of positive and negative control cell lines are included in supplementary material, Figure S3.

Digital images of immunostained TMA cores were created using a digital scanner (Aperio Scan Scope XT, Milton Keynes, UK). Cytoplasmic staining assessment in the invasive margins of primary breast tumours was carried out independently by two trained operators, blinded to outcome data, under the supervision of an experienced breast histopathologist (AMH) who also adjudicated discrepant scores. The level of agreement of the two scores was measured using Cohen's kappa coefficient. All scores were based upon intensity of staining and not the number of positive cells. Staining intensity was ranked based on a three‐tier ordinal categorical system used to rank the tumours based on intensity of cytoplasmic staining 15, 16, where 1 = weak staining; 2 = moderate, easily perceived staining; 3 = strong/intense staining, i.e. the scoring was based on staining intensity only.

### Statistical analyses

All immunohistochemical analyses followed REMARK guidelines 17. Statistical analyses evaluated the associations between protein expression and relevant clinical and pathological variables (e.g. ER/PR/HER2 status) using Fisher's exact test (categorical variables) and the Kruskal–Wallis test (continuous variables), before assessing prognostic and predictive associations with time‐to‐event data (time to first distant recurrence, time to first skeletal recurrence, time to first non‐skeletal recurrence) using Cox proportional hazards regression, the Kaplan–Meier estimate of the survival function and the log‐rank test. Time to first distant recurrence was defined as the time from the date of randomisation to the date of the distant recurrence. In analyses, other types of event were censored, e.g. if a local recurrence occurred prior to any distant recurrence, the patient would be censored at the date of the local recurrence. Time to first skeletal recurrence and first non‐skeletal recurrence were defined similarly. Time to first skeletal recurrence irrespective of all other previous recurrences was also investigated. Time to event analysis was first performed within treatment arms to identify prognostic associations with the biomarkers. The predictive heterogeneity of effect between treatment arms for time to distant events was assessed in multivariable analysis by including an interaction term in the Cox proportional hazard regressions for treatment arm and biomarker (while adjusting for systemic therapy plan, ER status and lymph node involvement). All significance tests were two‐sided and were designated significant at the 5% level.

## Results

### Proteomic identification of proteins specifically associated with breast cancer bone metastasis

Proteins identified in the ‘forward’ and ‘reciprocal’ labelling experiments (see supplementary material, Figure S1) were aligned and the most robust, highest quality data were extracted for analysis using the following stringent selection criteria: protein identified in both datasets; ≥2 so‐called razor+unique peptides assigned to identifications (clarifies assignment of protein identification); equal numbers of peptides and razor+unique peptides assigned to identifications. This resulted in 2006 proteins taken forward for analysis out of a total of 2999 identified in the complete dataset. (Full proteomic data are available on the publicly accessible database ORDA: https://orda.shef.ac.uk/.)

The aligned dataset was filtered further by using a 1.75‐fold cut‐off to distinguish change (up or down) in protein expression, resulting in 48 proteins upregulated in BM1 relative to PCC cells (see supplementary material, Table S1). These were prioritised for further study using literature evidence of relevance to bone metastasis, the magnitude of the differential expression fold change and evidence (where available) of non‐association with lung metastases, based on correlation with our other proteomic datasets that included a MDA‐MB‐231 variant, which specifically homes to lung 10, 11. This allowed us to identify proteins that were probably involved in breast cancer metastasis to bone. Consequently, four proteins had potential for further consideration: DOCK4 (fold‐change 2.7), SerpinB2 (fold‐change 15.6), cell‐division cycle protein 20 homolog (CDC20, fold‐change 3.7), and pericentrin (fold‐change 2.5). Although all four have published evidence linking them to breast cancer, we focused on DOCK4 for further investigation and clinical validation based on its published role in cell migration, including breast cancer cell migration, metastasis 18, 19, 20, 21 and tumour angiogenesis 14, processes known to be integral to dissemination of tumour cells and development of bone metastases. Moreover, DOCK4 functions as a guanine nucleotide exchange factor for the GTPase Rac1, a key regulator of motility 14, 19, 20 and localises at actin‐rich protrusions in migrating breast cancer cells 19, while SNPs within the promoter region of *DOCK4* have been detected in breast cancer 22.

### Confirmation of DOCK4 upregulation in BM1 cells

**Figure 1:**
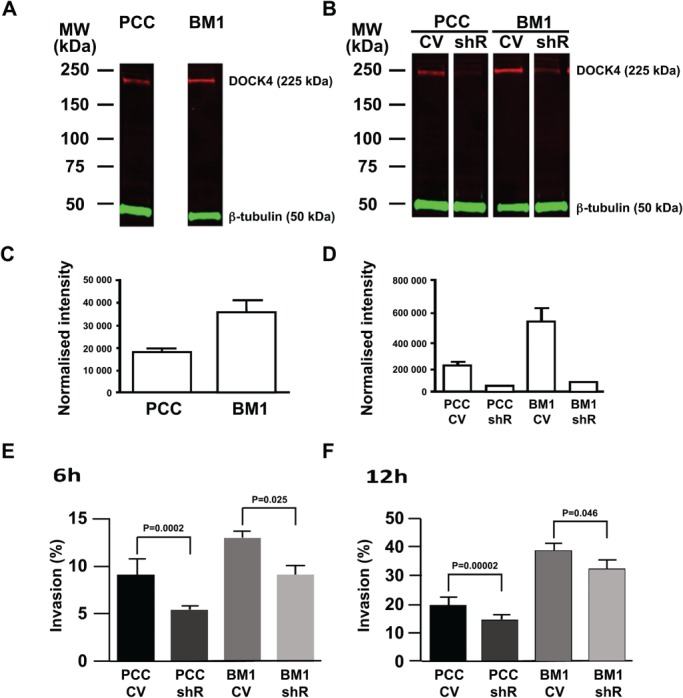
Expression of DOCK4 in BM and PCC cell lines and the effects of knockdown by shRNA. (A–C) DOCK4 was confirmed to be of higher expression in BM1 cell type by western blotting (two‐fold induction, *p* = 0.027); 81 and 88% knockdown of DOCK4 protein expression was achieved in PCC and BM1 cells, respectively. Representative whole gel lane images are shown. (E and F) Invasion/migration assay. The ability of PCC and BM1 cells to move through Matrigel™ matrix was assessed using a scratch‐wound assay. Significant differences between the control vector and DOCK4 knockdown cells were seen at the 6 and 12 h time points.

Analysis of DOCK4 expression levels by western blotting in the BM1 and PCC cell lines showed a two‐fold increase in DOCK4 expression within the bone‐homing BM1 cell line compared with PCC (Figure 1A,C). Increased expression of DOCK4 within BM1 cell total cell lysates was also confirmed using ECL‐based visualisation.

### DOCK4 is upregulated during breast cancer cell motility

There was significant knockdown of DOCK4 protein expression in the BM1 cell type following lentiviral delivery of DOCK4 shRNA compared with the empty vector (control vector) control (Figure 1B,D). In the wound healing assay, BM1 cells (control vector) had enhanced invasive and migratory ability compared with the PCC cells, with 13 and 39% wound closure at 6 and 12 h, respectively, compared with 9 and 20% for PCC at 6 and 12 h, respectively (Figure 1E,F). Invasion through Matrigel was reduced in cells with DOCK4 knockdown (shRNA) compared with control, with significant differences in wound closure observed for both cell types at 6 and 12 h, and with a greater effect seen at 6 h.

### Association between DOCK4 expression and tumour grade in local breast tumour array

**Figure 2:**
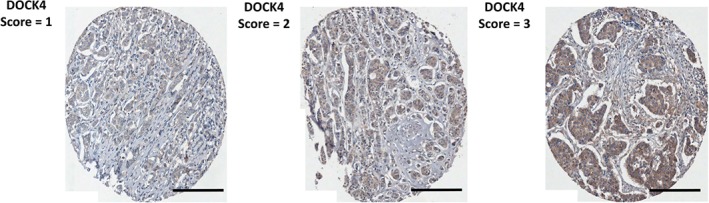
Examples of immunostaining for DOCK 4 in TMA cores from patients in the AZURE study. Examples of protein expression intensity scores for DOCK4 protein as assessed using immunohistochemistry and visualised at magnification of 20×. The scoring was based on the intensity of staining in the cytoplasmic compartment of the tumour cells only. Scale bar = 200 μm.

DOCK4 expression was initially assessed in a local breast tumour array of 345 unselected breast tumours (88% ductal, 9% lobular, 3% other) with patient data available on tumour grade (18% grade 1, 44% grade 2, 38% grade 3), ER and axillary lymph node involvement. Examples of typical staining patterns are shown in Figure 2. Analysis revealed a significant association between DOCK4 expression and histological tumour type (*p* = 0.002) and tumour grade (*p* = 0.004) with 86.4% grade 3 and 77.3% grade 2 ductal carcinomas expressing moderate/high DOCK4 (as opposed to 62.5% grade 1 carcinomas), but no association with ER status (positive versus negative, *p* = 0.185) or lymph node status (involved versus not involved, *p* = 0.15). These data suggest that DOCK4 expression was associated with tumour aggressiveness and further support our selection of DOCK4 for clinical validation in the AZURE patient cohort.

### Clinical validation of DOCK4 expression in breast cancer patients in the AZURE study

Independent scoring of stained TMAs by two trained operators (JW and SR) under the supervision of AMH yielded a Cohen's kappa coefficient value of 0.86, signifying excellent agreement. Possible associations between clinical outcomes and immunohistochemistry scores for DOCK4 were tested for 689 patients in the AZURE study (330 control arm, 359 zoledronate arm). Demographic data for these patients were similar to those of the whole AZURE population (Table 1). DOCK4 had no significant associations with age, lymph node involvement, ER status, tumour grade (although this approached significance, *p* = 0.062), menopausal status, systemic therapy, chemotherapy and statin use. HER2 status was not a mandated assessment but was available for 307 participants. DOCK4 was significantly associated with HER2 status, with a smaller proportion of patients with low DOCK4 being HER2 positive (*p* < 0.001). However, adjustment for HER2 status had no impact on subsequent statistical analyses in either control or zoledronate arms.

**Table 1:** Characteristics of the patients whose tissue was assessed on TMAs in this study (as at baseline on the AZURE study) and first DFS events

	DOCK4 dataset		Full AZURE trial population	
Characteristic	Zoledronate (*n* = 359)	Control (*n* = 330)	Zoledronate (*N* = 1681)	Control (*n* = 1678)
Age (years) median (range)	50 (26, 75)	51 (32, 79)	51 (20–89)	51 (21, 89)
Axillary lymph nodes – *n* (%)				
0	4 (1.2)	4 (1.2)	29 (1.7)	32 (1.9)
1–3	223 (67.6)	223 (67.6)	1041 (61.9)	1032 (61.5)
≥4	103 (31.2)	103 (31.2)	604 (35.9)	608 (36.2)
Tumour stage – *n* (%)				
T1	115 (32.0)	116 (35.2)	542 (32.2)	523 (31.2)
T2	196 (54.6)	163 (49.4)	851 (50.6)	867 (51.7)
T3	37 (10.3)	43 (13)	227 (13.5)	228 (13.6)
T4	11 (3.1)	8 (2.4)	58 (3.5)	59 (3.5)
Histological grade – *n* (%)				
1	26 (7.2)	23 (7.0)	145 (8.6)	140 (8.3)
2	144 (40.1)	130 (39.4)	731 (43.5)	708 (42.2)
3	187 (52.1)	174 (52.7)	765 (45.5)	787 (46.9)
Not specified		3 (0.9)		
Missing	2 (0.6)			
ER status – *n* (%)				
ER positive	255 (77.3)	255 (77.3)	1319 (78.5)	1316 (78.4)
ER negative	80 (22.3)	72 (21.8)	349 (20.8)	355 (21.2)
ER unknown	1 (0.3)	3 (9)	13 (0.8)	7 (0.4)
PR status – *n* (%)				
PR positive	126 (35.1)	102 (30.9)	725 (43.1)	698 (41.6)
PR negative	63 (17.5)	73 (22.1)	382 (22.7)	424 (25.3)
PR unknown	169 (47.1)	153 (46.4)	571 (34.0)	548 (32.7)
Missing	1 (0.3)	2 (0.6)		
HER2 status – *n* (%)				
HER2 positive	38 (10.6)	48 (12.7)	192 (11.4)	223 (13.3)
HER2 negative	106 (29.5)	84 (26.0)	648 (38.5)	603 (35.9)
HER2 unknown/not measured	209 (58.3)	197 (61.2)	831 (49.5)	843 (50.1)
Missing	6 (1.7)	1 (0.3)		
Menopausal status – *n* (%)				
Premenopausal	167 (46.5)	151 (45.8)	751 (44.7)	752 (44.8)
≤5 years since menopause	53 (14.8)	54 (16.4)	247 (14.7)	244 (14.5)
>5 years since menopause	112 (31.2)	97 (29.4)	519 (30.9)	522 (31.1)
Menopausal status unknown	27 (7.5)	28 (8.5)	164 (9.8)	160 (9.5)
				
Planned systemic therapy – *n* (%)				
Endocrine therapy alone	24 (6.7)	17 (5.2)	76 (4.5)	74 (4.5)
Chemotherapy alone	79 (22.0)	72 (21.8)	362 (21.5)	360 (21.5)
Endocrine therapy plus chemotherapy	256 (71.3)	241 (73.0)	1243 (73.9)	1243 (74.1)
Type of chemotherapy – *n* (%)				
Anthracyclins	328 (91.4)	307 (93.0)	1567 (97.6)	1564 (97.6)
Taxanes	48 (13.4)	41 (12.4)	390 (24.3)	385 (24.0)
Timing of chemotherapy				
Neoadjuvant	14 (3.3)	11 (3.3)	104 (6.5)	104 (6.5)
Postoperative	345 (96.7)	319 (96.7)	1501 (93.5)	1499 (93.5)
Statin use – *n* (%)	19 (5.3)	15 (4.5)	97 (5.8)	101 (6.0)
Type of first DFS event – *n* (%)				
Locoregional recurrence	26 (7.2)	17 (5.2)	79 (4.7)	78 (4.7)
Distant recurrence	68 (18.9)	74 (22.4)	332 (19.8)	341 (20.3)
Distant and locoregional recurrence	5 (1.4)	3 (0.9)	18 (1.1)	21 (1.3)
Death without prior recurrence	11 (3.1)	13 (3.9)	53 (3.2)	44 (2.6)
First distant recurrence is non‐skeletal – *n* (%)				
	46 (12.8)	28 (8.5)	194 (11.5)	165 (9.8)
First distant recurrence includes skeletal and other – *n* (%)				
	27(7.5)	49 (14.8)	156 (9.3)	197 (11.7)
First distant recurrence is skeletal only – *n* (%)				
	16 (4.5)	38 (11.5)	97 (5.8)	140 (8.3)

Non‐skeletal, first distant recurrence event does not include any skeletal component; skeletal and other, first distant recurrence event reported includes both skeletal and other sites of metastasis, as well as skeletal only; skeletal only, first distant recurrence event only skeletal ‐ this group is a subset of those classified as skeletal and other. PR, progesterone receptor.

#### AZURE patients: association of DOCK4 expression with distant event recurrence

##### Control arm

**Figure 3:**
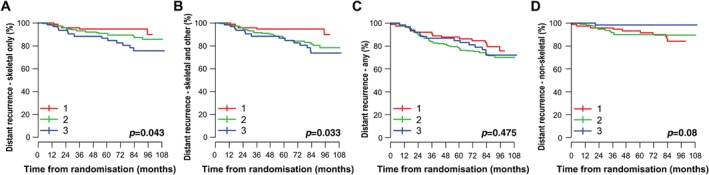
Association of DOCK4 with DFS events. Kaplan–Meier estimates of the relationship between expression of DOCK4 and (A) skeletal only DFS events (where no other distant event was recorded at the same time) , (B) skeletal and other DFS events (where other distant events may have been recorded at the same time), (C) any DFS events and (D) non‐skeletal DFS events in patients in the control arm of the AZURE trial (*n* = 434). P value is from the log‐rank test for testing equality of survival Q15 functions.

Initial analyses considered possible associations between DOCK4 expression (scored as 1, 2 or 3) and DFS for distant recurrence components of DFS. Although we found no statistically significant association between DOCK4 and the first event in non‐skeletal sites, taken as a group (*p* = 0.08, Figure 3D) or in any distant site (*p* = 0.475, Figure 3C), higher DOCK4 was significantly associated with an increased risk of developing the first event as skeletal recurrence whether only in bone (*p* = 0.043, Figure 3A) or in bone and other distant sites concurrently (*p* = 0.033, Figure 3B).

**Figure 4:**
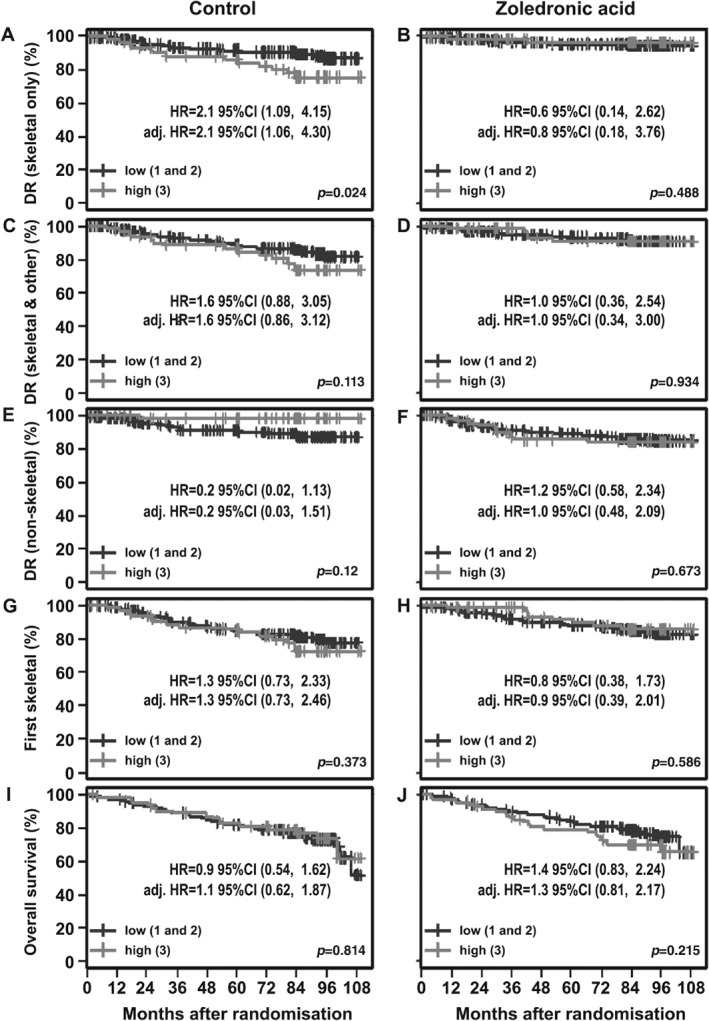
Univariate associations of distant recurrence outcomes with biomarker expression in control and zoledronate arms (estimates are from Cox proportional hazards regressions). Kaplan–Meier estimates of the survival function for time to distant recurrence (DR) and overall survival for control and zoledronate arms. Numbers 1 to 3 refer to the DOCK4 staining intensity scores. These were dichotomised, i.e. DOCK4 low (1 and 2); DOCK4 high (3). Comparisons shown to be significant were also significant in analyses adjusting for the effect of systemic therapy plan, ER status and lymph node involvement. (A and B) Skeletal only, (C and D) skeletal and other, (E and F) non‐skeletal, (G and H) first skeletal irrespective of whether other distant events have occurred previously (i.e. bone metastasis‐free survival). (I and J) Overall survival. *P* values refer to the log‐rank test. For definitions of non‐skeletal, skeletal and other and skeletal only see legend to Table 1.

Subsequently, dichotomised TMA expression scores were used where high DOCK4 expression (score of 3) was compared with low DOCK4 expression (score of 1 or 2). Using these categories, Kaplan–Meier estimates of the survival function for time to distant recurrence confirmed that high DOCK4 is significantly prognostic for first distant recurrence involving bone only (HR 2.1; 95%CI 1.09, 4.15, *p* = 0.024, Figure 4A). For the first distant event involving both skeletal and other site(s) concurrently, although the same trend was observed, the association did not reach significance (HR 1.6; 95%CI 0.88, 3.05, *p* = 0.113, Figure 4C).

Where the first distant event was in non‐skeletal sites (taken as a group), the corresponding dichotomised analyses (Figure 4E) suggested a non‐significant reduced risk of non‐skeletal events with high DOCK4 (HR = 0.2; 95%CI 0.03, 1.51, *p* = 0.12). In the dichotomised analyses, we found no association between high DOCK4 and first skeletal event, whether or not other distant events had occurred first (HR = 1.3; 95%CI 0.73, 2.33, *p* = 0.373), suggesting that the inclusion of metastasis in other sites, before eventual spread to bone, counters the significant association seen with the bone‐only analyses.

##### Zoledronate arm

In the zoledronate arm, dichotomised analyses revealed no association between DOCK4 and bone‐only distant events (HR 0.6; 95%CI 0.14, 2.62, *p* = 0.488, Figure 4B), i.e. the increased risk of bone‐only first event posed by high DOCK4 in the control arm was abolished by zoledronate. This suggests that DOCK4 may act as a predictive biomarker for the prevention of bone metastases by zoledronate. This was confirmed by examining Kaplan–Meier plots for DOCK4 high and DOCK4 low comparing control and zoledronate patients for the various events (see supplementary material, Figure S5). Figure S5B clearly shows the reduction in bone metastasis‐only risk as a first event (HR 0.1; 95%CI 0.03, 0.5, *p* = 0.003) in patients with high DOCK4 treated with zoledronate.

For the first event involving bone and other sites concurrently, there was no significant association seen in the zoledronate group (Figure 4D).

#### Cox proportional hazards regressions adjusted for systemic therapy plan, ER status, HER2 status and lymph node involvement

##### Control arm

Adjusted analyses (Table 2) confirmed that high DOCK4 expression was significantly prognostic for skeletal‐only events (HR 2.13, 95%CI 1.06, 4.30, *p* = 0.034). Similar analyses for first recurrence events involving the skeleton and other site(s) concurrently showed no significant association (HR 1.63, 95%CI 0.86, 3.12, *p* = 0.137; Table 2). No significant association was found in these adjusted analyses for non‐skeletal distant events when assessed collectively. Analysis of relationships to other individual metastatic sites was not possible with this dataset.

**Table 2:** Cox regression analysis for associations between protein immunohistochemistry and distant recurrence events by AZURE trial arm

		Standard treatment				Standard treatment + zoledronate			
		No. at risk	No. events	HR(95%CI)	*P* value	No. at risk	No. events	HR(95%CI)	*P* value
Non‐skeletal distant recurrence	Unadjusted	330	28	0.154 [0.021,1.131]	0.066	359	46	1.162 [0.577,2.342]	0.674
	Adjusted	329	28	0.201 [0.027,1.506]	0. 118	353	46	0.999 [0.478,2.086]	0.997
Distant recurrence including skeletal	Unadjusted	330	49	1.642 [0.883,3.052]	0.117	359	27	0.96 [0.363,2.535]	0.934
	Adjusted	329	49	1.634 [0.855,3.121]	0.137	353	26	0.999 [0.336,2.967]	0.998
Skeletal‐only distant recurrence	Unadjusted	330	38	2.121 [1.085,4.148]	0.028	359	16	0.595 [0.135,2.619]	0.493
	Adjusted	329	38	2.133 [1.058,4.304]	0.034	353	16	0.812 [0.176,3.756]	0.79
Bone metastasis at any time	Unadjusted	330	62	1.302 [0.728,2.328]	0.374	359	49	0.81 [0.38,1.728]	0.586
	Adjusted	329	62	1.344 [0.734,2.46]	0.338	353	47	0.882 [0.387,2.012]	0.765

The reference category in each multivariable model is DOCK4 low (1 or 2). Comparisons shown to be significant are also significant in analyses adjusting for the effect of systemic therapy plan, ER status, HER2 status and lymph node involvement. Time to first bone metastasis is defined as time to the first skeletal distant recurrence, irrespective of whether other distant recurrence has occurred earlier (i.e. bone metastasis‐free survival). For definitions of non‐skeletal, skeletal plus other and skeletal only, see legend to Table 1.

##### Zoledronate arm

The significant prognostic effect of DOCK4 for skeletal‐only metastases seen in the control arm patients was not observed in the zoledronate arm (HR 0.812, 95%CI 0.18, 3.76, *p* = 0.790; Table 2), suggesting again that the increased risk for skeletal metastasis in patients with high DOCK4 levels at baseline may be counteracted by treatment with zoledronate. This was tested formally by including an interaction term in the Cox proportional hazards regressions for treatment arm and DOCK4 level for time to first event, skeletal only. These analyses suggested a predictive effect for treatment with zoledronate (HR 0.12 95%CI 0.03, 0.56; likelihood ratio test *p* = 0.063; HR < 1 indicates improvement with zoledronate), although this did not reach significance.

#### Overall survival and menopausal status

As shown in Figure 4I,J and supplementary material, Figure S5I,J, although there appeared to be a trend towards high DOCK4 producing worse outcome, DOCK4 expression level did not impact significantly on overall survival in either the control or the zoledronate arms. When postmenopausal and premenopausal patients were analysed separately, although similar associations with bone metastasis to the full group were observed, there was a loss of statistical significance and this may be due to the smaller numbers involved.

## Discussion

Breast cancer bone metastasis causes significant morbidity and biomarkers that can predict the development of bone metastases are badly needed. In this translational study, SILAC‐MS‐based comparison of bone‐homing and non‐homing cell variants and functional *in vitro* work, coupled with rigorous clinical validation, enabled us to identify DOCK4 as a potential biomarker for this purpose.

Our discovery science used MDA‐MB‐231 and its bone‐homing variant BM1. The MDA‐MB‐231 cell line is regarded as the ‘gold standard’ for this type of research as it was derived from a pleural effusion of a breast cancer patient with widespread tumour metastasis, many years after resection of the primary tumour 23. Furthermore, the validity of this approach has already been proven in our previous work in which the importance of the proteins CAPG and GIPC1 as biomarkers was discovered and subsequently clinically validated 10. Here we focused on DOCK4, a key guanine nucleotide exchange factor regulating the activation of the small GTPase Rac1 19, 20, 24, 25. DOCK4‐mediated activation of Rac1 has been demonstrated to promote actin reorganisation and the formation of lamellipodia at the leading edge of breast cancer cells 19, as well as the formation of lateral filopodia and blood vessel lumen morphogenesis within tumour angiogenesis 14.

DOCK4 was expressed in parental MDA‐MB‐231 cells (PCC), but was more abundant in the bone‐homing variant (BM1). DOCK4 knockdown inhibited the migration of both PCC and BM cell lines, with inhibition being greater at 6 h than 12 h post‐assay initiation, suggesting that DOCK4‐mediated cell invasion may be important in the earlier stages of breast cancer cell migration. However, enhanced cell migration and invasiveness resulting from high DOCK4 expression is only one aspect of the bone‐homing cells that makes them bone homing. In particular, c‐MAF targets and other proteins elevated in the bone‐homing cells might drive the bone‐homing phenotype as well as DOCK4. Our observation that, in patients, high DOCK4 is specifically associated with first distant metastasis in bone, may be linked to these factors, including those in the bone microenvironment. Notably, in this regard, the specific association of high DOCK4 with bone metastasis at any time is lost once metastasis has occurred elsewhere. This presumably indicates the substantially altered metastatic environment influencing bone metastasis once non‐bone metastases have occurred. Association with bone as the first metastatic site is lost in zoledronate‐treated patients, suggesting that zoledronate treatment reduces the risk of bone metastasis to a level similar to that in non‐high DOCK4 patients.

This work has shown that DOCK4 has a similar prognostic and predictive profile to CAPG and GIPC1 for the prognosis of skeletal‐only relapse within control arm patients. DOCK4 was also similar to the previously discovered markers in not being predictive of non‐skeletal recurrence events within control arm patients. Treatment with zoledronate abolished the association of high levels of all three of these proteins in the development of skeletal‐only metastasis.

DOCK4 expression is induced by the cytokine TGFβ acting via the Smad pathway, and this is a key step in TGFβ's pro‐metastatic effect 21. GIPC1 is also a key scaffolding protein that functions to transmit signals from the TGFβ receptor and its coreceptor endoglin to downstream Smad phosphorylation 26. TGFβ is a regulator of numerous steps within metastasis, including intravasation, extravasation and cancer cell survival at distant organ sites 27. The transcription factor c‐MAF has recently been identified as a key regulator of breast cancer bone metastasis 28, 29. c‐MAF expression is induced by TGFβ and a recent patent application showed that DOCK4 expression correlates with MAF expression within primary tumours 30. Examination of our quantitative proteomic dataset identified 36 proteins also present in the c‐MAF gene set (a panel of 109 genes in total). Within the 36 proteins quantified by proteomics, 15 proteins displayed the same change (increase or decrease of expression within bone‐homing cells compared with parental cells) as the relevant gene transcripts in response to c‐MAF expression. DOCK4 may therefore be a component of a protein panel that responds to elevated c‐MAF expression within bone‐homing breast cancer cells.

As well as being a prognostic biomarker, our data also provide evidence that DOCK4 is a potential predictive biomarker in terms of the treatment effect of zoledronate for bone as the first metastatic site, as the addition of zoledronate appears to reduce the risk of patients with high DOCK4 levels to that of patients with lower DOCK4 levels. There was also a substantial HR in favour of a treatment effect when an interaction term was included in the Cox proportional hazards regression, although this fell short of statistical significance and additional testing in a further patient cohort is needed before DOCK4 can be confirmed as a predictive biomarker.

Our current study is supported further through analysis of *DOCK4* gene expression within a publicly available database where high *DOCK4* transcript levels predicted distant bone metastatic spread of breast cancer (see supplementary material, Figure S4).

Interestingly, DOCK4 was not prognostic for DFS in terms of metastasis at non‐skeletal sites, taken as a group (*n* = 28). Indeed, data for the control arm in Figure 4 and Table 2 (and see supplementary material, Figure S5) suggest that high DOCK4 may be associated with a reduction in the occurrence of non‐skeletal metastasis, an effect that is not seen in the zoledronate arm. Although zoledronate is advantageous in terms of preventing skeletal metastasis in DOCK4 high patients, it may also remove a potentially advantageous effect for non‐skeletal metastases. Although our data do not point to any particular mechanistic explanation for this effect, it is possible that zoledronate may have effects other than on the skeleton and, in this respect, we note the negative impact of zoledronate on overall survival in premenopausal women recently reported, where in non‐postmenopausal patients with MAF‐positive tumours, zoledronate was associated with worse invasive DFS and overall survival 28. Clearly, these factors need to be borne in mind in the consideration of DOCK4 as a predictive biomarker for zoledronate response in bone metastasis prevention. Because our analyses included non‐skeletal sites as a single group, they do not exclude the possibility of DOCK4 association with a less common site for metastatic spread than the skeleton.

There are some limitations to the current study. Although the number of patients available for analysis from the AZURE study considerably exceeds that required for statistical powering, there is currently no equivalent independent sample set available for further validation. Also, breast cancer metastasis to bone involves numerous autocrine and paracrine signalling events 31, and these, and the directionality and recruitment of key signalling pathways that migration involves, are clearly not replicated within the invasion assay used in the current study. More complex tools to assess this are not currently available.

Further studies of the mechanistic role of DOCK4 in breast cancer bone metastasis and implications for pharmacological inhibition are justified by our work.

## Author contributions statement

JW, SW, KM, RG, HT, MO, MD, SR, AH, PS, VS GM, RC and JB conceived and/or carried out the experiments. KM, VS and GM contributed the data in the training cohort. JW, DC, SW, HM, AH, MD, PS, VS, GM, RC and JB analysed the data. All authors were involved in writing the manuscript and had final approval of the submitted version.

**SUPPLEMENTARY MATERIAL ONLINE**

**Supplementary materials and methods**

**Figure S1.** Schematic showing the key steps in the SILAC proteomic approach used

**Figure S2.** Testing antibody specificity by immunostaining of FFPE cells

**Figure S3.** Testing antibody specificity by western blotting of cell lysates

**Figure S4.** Gene expression analysis of *DOCK4* expression and time to bone metastasis

**Figure S5.** Univariate associations of distant recurrence outcomes with biomarker expression for DOCK4 low and DOCK4 high

**Table S1.** Proteomic results for upregulated proteins within the SILAC comparison of parental (PCC) and bone‐homing (BM1) MDA‐MB‐231 cells

## Supporting information


**Supplementary materials and methods**

**Figure S1**. Schematic showing the key steps in the SILAC proteomic approach used. MDA‐MB‐231 (MDA231) and bone‐homed variant (BM) cell lines were incubated with media containing ‘heavy’ and ‘light’ isotopically‐labelled amino acids arginine (R) and lysine (K). The heavy medium contained the stable isotopes of [^12^C_6_, ^14^N_4_]‐L‐Arginine and [^12^C_6_, ^14^N_2_]‐L‐Lysine. Two experiments were performed involving ‘forward’ and ‘reverse’ labelling (reciprocal labelling) in which the cell lines were incubated in both media types and then combined in heavy: light pairs in a 1:1 ratio (based on extracted protein assay) prior to separation by molecular weight using 1D electrophoresis. The entire lane of gel‐separated proteins was cut into equal slices (*n* = 10). The proteins in each gel slice were reduced to peptides by enzymatic digestion (using trypsin) and were further separated using high‐performance liquid chromatography (HPLC) and analysed using high‐resolution mass spectrometry (MS).
**Figure S2**. Testing antibody specificity by immunostaining of FFPE‐cells. (A) Western blotting confirmation of DOCK4 knockdown in DOCK4 miRNA transfected MDA‐MB‐231 cells. (B) Representative microscopy fields from FFPE bone‐homing MDA‐MB‐231 cells (BM1) stably transfected with either control lentivirus (Control vector) or DOCK4‐miRNA‐expressing lentivirus (DOCK4‐miRNA) and subjected to immunostaining using the Bethyl anti‐DOCK4‐antibody and a DAB colour reaction with Haematoxylin counterstaining. Two representative fields are shown for each cell‐type. (C) Quantification of mean cellular DAB staining level across five replicates of each cell‐type (mean ± SEM, *n* = 5).
**Figure S3**. Testing antibody specificity by Western blotting of cell‐lysates. (A) Full gel length ECL images of Western blots of 50 μg total cell lysate from PCC‐cells
(lane 1) and BM1 cells (lane 2). Western blotting was performed using the Abcam anti‐DOCK4 antibody (ab56743) as described. A tubulin loading control is also shown. Quantification of the normalised DOCK4 band intensity is depicted in the attached histogram (*n* = 3 replicate gels, mean band intensity ± SEM). (B) Full gel length ECL images of Western blots of 50 μg total cell lysate from PCC (lane 1) and BM1‐cells (lane 2) probed with the Bethyl anti‐DOCK4 antibody. A tubulin loading control included. Histogram depicts the quantification of the normalised DOCK4 band intensity (mean ± SEM, *n* = 3, replicate gels).
**Figure S4**. Gene expression analysis of *DOCK4* expression and time to bone metastasis. Time to bone metastasis analysis of *DOCK4* expression level within breast cancer patients from Wang, *et al.et al* [32] with high (>8.53) and low (<8.53) levels of *DOCK4* expression.
**Figure S5**. Univariate associations of distant recurrence outcomes with biomarker expression for DOCK4 low and DOCK4 high. (Estimates are from Cox proportional hazards regressions). Kaplan‐–Meier estimates of the survival function for time to distant recurrence (DR) and overall survival for control and zoledronate arms for dichotomised DOCK4 low (1 and 2) and high (3). Numbers 1 to 3 refer to the DOCK4 staining intensity scores. Comparisons shown to be significant are also significant in analyses adjusting for the effect of systemic therapy plan, ER status and lymph node involvement. (a,b): Skeletal only; (c,d): Skeletal and other; (e,f): Non‐skeletal; (g,h): First skeletal irrespective of whether other distant events have occurred previously (i.e. bone metastasis‐free survival). (i,j): Overall Survival (OS). *P*‐values refer to the logrank test. For definitions of non‐skeletal, skeletal and other and skeletal only see legend to Table 1.

**Table S1.** Proteomic results for up‐regulated proteins within the SILAC comparison of parental (PCC) and bone‐homing (BM1) MDA‐MB‐231 cells.
