# Supplementary material for: Identification and validation of DOCK4 as a potential biomarker for risk of bone metastasis development in patients with early breast cancer
Source: J Pathol. 2019 Jan 25;247(3):381–91. doi: 10.1002/path.5197 (PMC6618075; doi:10.1002/path.5197)
Supplement: Supplementary file 1 — Supplementary materials and methods [file PATH-247-381-s001.docx]

**Identification and validation of DOCK4 as a potential biomarker for risk of bone metastasis development in patients with early breast cancer**

Westbrook JA *et al*. *J Pathol* 2018 (DOI: 10.1002/path.5197)

**Supplementary materials and methods**

Reference numbers refer to the main text list

***Cell culture and stable isotope labelling by amino acids in cell culture (SILAC)***

The human breast cancer cell line MDA-MB-231 (PCC, parental control cells, obtained originally from ATCC) and a bone-homing variant (BM1, bone metastatic cells) [11] (the latter supplied by Professor Joan Massagué, Sloan-Kettering Institute, New York, NY, USA), were used in a ‘classical’ SILAC experiment [12,13]. Quarterly mycoplasma checks and annual STR profiling confirmed cell provenance. PCC and BM1 cells were cultured in ‘heavy’ SILAC medium and ‘light’ medium for ~10 doublings to ensure >95% isotope label incorporation (heavy media) and all cells were used within 10 doublings. ‘Heavy’ SILAC media consisted of DMEM containing l-arginine (^13^C_6_, ^15^N_4_) and l-lysine (^13^C_6_, ^15^N_2_) (R10K8, DMEM-15, Dundee Cell Products, Dundee, UK) supplemented with dialysed (10 kDa molecular weight cut-off [MWCO]) FBS (D-FBS100, Dundee Cell Products). ‘Light’ media was DMEM but without the heavy isotopes (R0K0) and including 10% (v/v) dialysed (10 kDa MWCO) FBS. Essentially, two experiments were performed involving ‘forward’ and ‘reverse’ labelling (i.e. reciprocal labelling) in which the cell lines were incubated in both media types and then combined in heavy:light pairs in a 1:1 ratio (based on extracted protein amount) prior to separation by molecular weight using 1-DE. For proteomic analysis, cells were extracted for total protein content using Laemmli buffer (S-3401 Sigma-Aldrich, Poole, UK).

***LC-MS/MS***

Equal amounts of protein (40 µg) from light and heavy labelled samples were combined, reduced, alkylated and separated on a 1D gel. LC-MS/MS was performed by Dundee Cell Products. Ten slices of the gel-resolved proteins were cut and proteins were digested to peptides using trypsin. Tryptic peptides were separated using a nanoflow LC-System coupled to an LTQ-Orbitrap mass spectrometer (ThermoFisher Scientific, Warrington, UK). Survey full scan MS spectra (m/z 300–1700) were acquired in the Orbitrap with a resolution of 60 000 at m/z 400 after accumulation of 1 000 000 ions. The five most intense ions from the preview survey scan delivered by the Orbitrap were sequenced by collision-induced dissociation (normalised collision energy = 40%) after accumulation of 5000 ions concurrently to full scan acquisition in the Orbitrap. Data were acquired using Xcalibur software.

***Quantitation and bioinformatics analysis***

Quantitation was performed using the software Max Quant (http://www.maxquant.org/downloads.htm), with peptide ratios calculated for each arginine- and/or lysine-containing peptide as the peak area of labelled arginine/lysine divided by the peak area of non-labelled arginine/lysine for each single-scan mass spectrum. Peptide ratios for all arginine- and lysine-containing peptides sequenced for each protein were averaged. Data output from Max Quant were analysed further using Excel and R (v. 3.2, <http://www.r-project.org/>) to select differentially expressed proteins for consideration as candidates for further verification and validation.

**Western blotting**

The differential expression of DOCK4, and confirmation of DOCK4 knockdown, was assessed in cell lysates using western blotting and an infrared immunodetection system (LI-COR Biosciences, Lincoln, NE, USA) as well as by ECL (Promega, Southampton, UK). The primary antibodies used were: DOCK4 (Abcam, Cambridge, UK; ab56743, mouse monoclonal, 0.1 µg/ml, lot: GR133602-1); beta-tubulin loading control (Abcam; ab6046, rabbit polyclonal, 1:5000 dilution, lot: GR3199392-1). Secondary antibodies were: #925-68070 IRDye 680RD goat anti-mouse IgG (H+L), LI-COR Biosciences, 1:5000 dilution, for the detection of DOCK4 in the 700 nm channel (red); #925-32211 IRDye 800CW goat anti-rabbit IgG (H+L), LI-COR Biosciences, 1:5000 dilution, for the detection of beta-tubulin in the 800 nm channel (green). Normalised densitometric data from six replicate runs of the immunoprobed samples were tested for significance using Student’s *t*-test. For ECL the secondary antibodies used were: goat anti-mouse-HRP (Abcam, ab6789) and goat anti-rabbit-HRP (Abcam, ab 6721) both used at 1:2500 dilution followed by visualisation with ECL reagent (Promega).

**Immunohistochemistry**

Bone homing MDA-MB-231 cells (BM1) were subjected to lentiviral transfection of either control plasmid (control vector) or plasmid expressing anti-DOCK4 miRNA (DOCK4 miRNA) as described in the main text, Materials and methods. Cells were cultured in DMEM + 10% (v/v) FBS until confluent and then harvested by trypsinisation, cells pelleted at 1000 ×*g* and the cell pellet washed twice with PBS. Cell pellets were then resuspended in 10% formalin in neutral buffered saline and fixed for 48 h at 4 °C. In preparation for sectioning, 100 µl 10% (w/v) molten agarose was allowed to set in Eppendorf tubes, forming an agarose plug. Following fixation of the cells in 10% (v/v) formalin in neutral buffered saline they were pelleted at 1000 ×*g* and then resuspended in 300 µl (10% w/v) molten agarose. Cells in molten agarose were introduced into the preprepared Eppendorf tubes with 100 µl agarose plugs and allowed to set overnight at 4 °C. The next day cell pellets were progressively dehydrated in graded alcohol followed by xylene and then finally molten wax treatment. Cell pellets were embedded in wax blocks and sectioned at 5 µm onto Superfrost Slides (Menzel-Gläser, supplied by VWR, Lutterworth, UK), then dried for approximately 48 h at 37 °C. Sections were dewaxed in xylene and rehydrated in graded alcohol and then endogenous peroxidase was blocked with 10% (v/v) hydrogen peroxide. Antigen retrieval was accomplished using citrate buffer (pH 6.0) in a microwave oven for 20 min. Sections were blocked in 10% (v/v) goat serum and then primary antibody (Bethyl Laboratories, Montgomery, TX, USA: rabbit anti-DOCK4 antibody, cat no: A302-263A, lot: A302-261-A-1) added overnight at a dilution of 1:100. Sections were developed using biotinylated secondary antibody (Vector Labs, Peterborough, UK) at a dilution of 1:200 for 1 h. Sections were rinsed and developed using the Vector Labs ABC kit (cat no: PK-6100, standard) for 30 min; DAB was applied for 1 min and then sections were rinsed and counterstained with Gill’s haematoxylin. Sections were then rehydrated with graded alcohols and mounted using DPX. Slides were scanned using a Pannoramic 250 slide scanner (3DHistech, Budapest, Hungary) and images analysed for DAB staining intensity within the QuPath software program.

**Testing antibody specificity: western blotting**

Total cell lysates from bone-homing MDA-MB-231 cells (BM1) and parental controls (PCC) were prepared in 1× Laemmli sample buffer. In addition, cell lysates were also prepared from bone-homing (BM1) cells following lentiviral transfection of either control vector or DOCK4 miRNA. All cell lysates were assayed for protein content using the Bio-Rad RC-DC protein assay kit (Bio-Rad, Deeside, UK). Aliquots (50 µg) of total cell protein were loaded onto each lane of a 10-lane Bio-Rad Mini-Protean 4–20% TGX^TM^ SDS-PAGE gel and blotted onto a nitrocellulose membrane. Following blocking of membranes overnight at 4 °C with 5% (w/v) milk powder in PBS they were probed with either: Abcam anti-DOCK4 (ab56743) at a dilution of 1:3400 or the Bethyl anti-DOCK4 antibody (A302-263A) at a 1:2000 dilution for 1 h. Blots were washed and secondary antibody applied (for Abcam ab56743, a mouse secondary was applied and for Bethyl anti-DOCK4 a rabbit secondary; both secondaries were used at a 1:2500 dilution) for 1 h. Blots were then washed with PBS-tween and developed with ECL-reagent (Promega, cat. no: W1001) prior to exposure to X-ray film.

**Gene-expression analysis for *DOCK4***

Gene expression data published by Wang *et al* [32] were obtained from the breastCancerVDX [33] Bioconductor package, and sample metadata from the GEO submission (GSE2034) was used to determine which samples had metastasis to the bone. A recursive partitioning (RP) analysis [34] was performed to determine if the samples can be split into two groups based on the expression data of DOCK4. Difference in the time to metastasis in the two groups was then assessed using the “survival” R package [35].
