# Supplementary material for: Identification and validation of DOCK4 as a potential biomarker for risk of bone metastasis development in patients with early breast cancer
Source: J Pathol. 2019 Jan 25;247(3):381–91. doi: 10.1002/path.5197 (PMC6618075; doi:10.1002/path.5197)
Supplement: Supplementary file 2 — Figure S1. Schematic showing the key steps in the SILAC proteomic approach used. MDA‐MB‐231 (MDA231) and bone‐homed variant (BM) cell lines were incubated with media containing ‘heavy’ and ‘light’ isotopically‐labelled amino acids arginine (R) and lysine (K). The heavy medium contained the stable isotopes of [12C6, 14N4]‐L‐Arginine and [12C6, 14N2]‐L‐Lysine. Two experiments were performed involving ‘forward’ and ‘reverse’ labelling (reciprocal labelling) in which the cell lines were incubated in both media types and then combined in heavy: light pairs in a 1:1 ratio (based on extracted protein assay) prior to separation by molecular weight using 1D electrophoresis. The entire lane of gel‐separated proteins was cut into equal slices (n = 10). The proteins in each gel slice were reduced to peptides by enzymatic digestion (using trypsin) and were further separated using high‐performance liquid chromatography (HPLC) and analysed using high‐resolution mass spectrometry (MS). Figure S2. Testing antibody specificity by immunostaining of FFPE‐cells. (A) Western blotting confirmation of DOCK4 knockdown in DOCK4 miRNA transfected MDA‐MB‐231 cells. (B) Representative microscopy fields from FFPE bone‐homing MDA‐MB‐231 cells (BM1) stably transfected with either control lentivirus (Control vector) or DOCK4‐miRNA‐expressing lentivirus (DOCK4‐miRNA) and subjected to immunostaining using the Bethyl anti‐DOCK4‐antibody and a DAB colour reaction with Haematoxylin counterstaining. Two representative fields are shown for each cell‐type. (C) Quantification of mean cellular DAB staining level across five replicates of each cell‐type (mean ± SEM, n = 5). Figure S3. Testing antibody specificity by Western blotting of cell‐lysates. (A) Full gel length ECL images of Western blots of 50 μg total cell lysate from PCC‐cells (lane 1) and BM1 cells (lane 2). Western blotting was performed using the Abcam anti‐DOCK4 antibody (ab56743) as described. A tubulin loading control is also shown. Quantific [file PATH-247-381-s002.docx]

**Identification and validation of DOCK4 as a potential biomarker for risk of bone metastasis development in patients with early breast cancer**

Westbrook JA *et al*. *J Pathol* 2018 (DOI: 10.1002/path.5197)


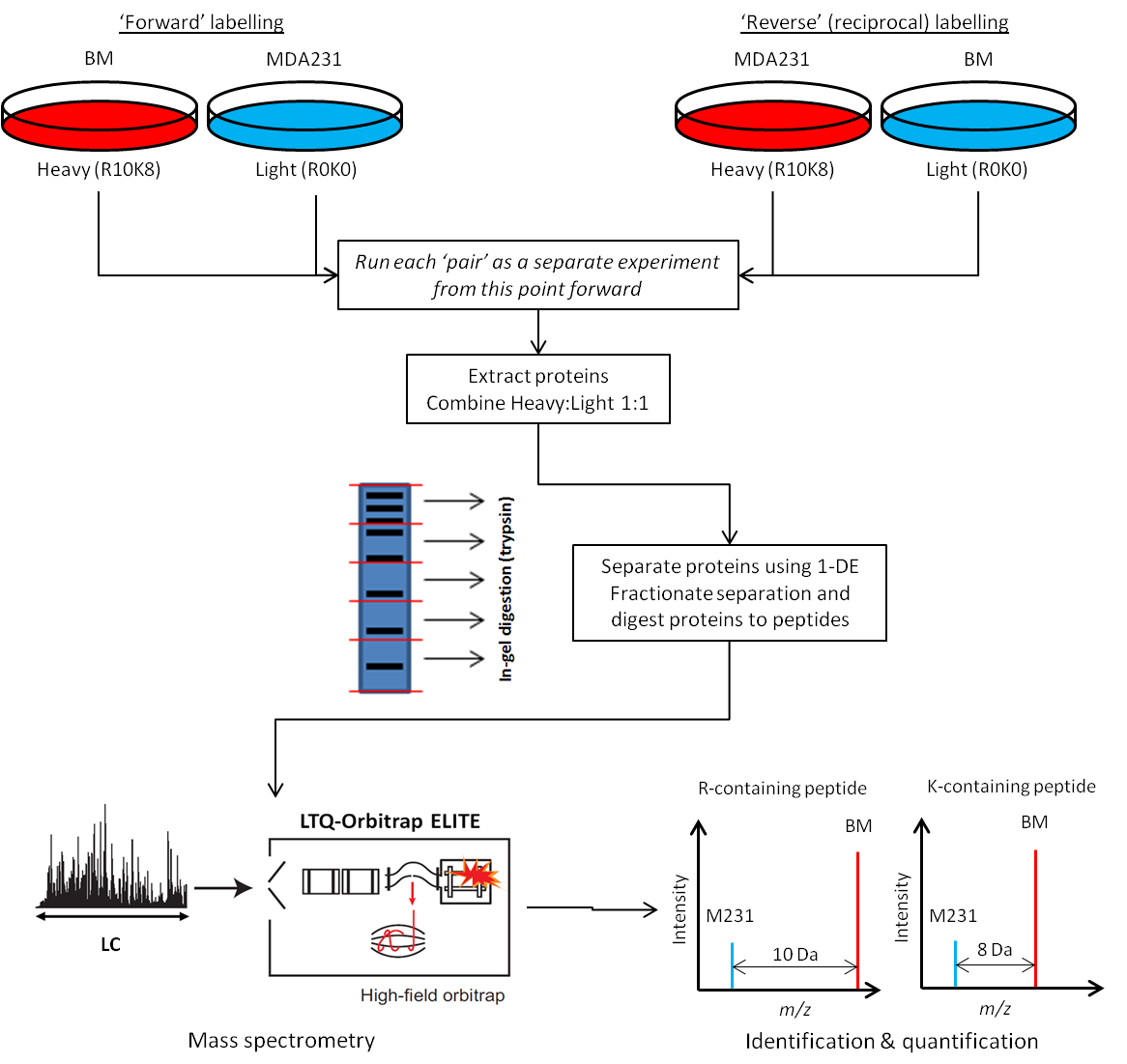


**Figure S1.** **Schematic showing the key steps in the SILAC proteomic approach used.**
MDA-MB-231 (MDA231) and bone-homed variant (BM) cell lines were incubated with media containing ‘heavy’ and ‘light’ isotopically labelled amino acids arginine (R) and lysine (K). The heavy medium contained the stable isotopes of [^12^C_6_, ^14^N_4_]-l-arginine and [^12^C_6_, ^14^N_2_]-l-lysine. Two experiments were performed involving ‘forward’ and ‘reverse’ labelling (reciprocal labelling) in which the cell lines were incubated in both media types and then combined in heavy:light pairs in a 1:1 ratio (based on extracted protein assay) prior to separation by molecular weight using 1D electrophoresis. The entire lane of gel-separated proteins was cut into equal slices (*n* = 10). The proteins in each gel slice were reduced to peptides by enzymatic digestion (using trypsin) and were further separated using HPLC and analysed using high-resolution mass spectrometry (MS).


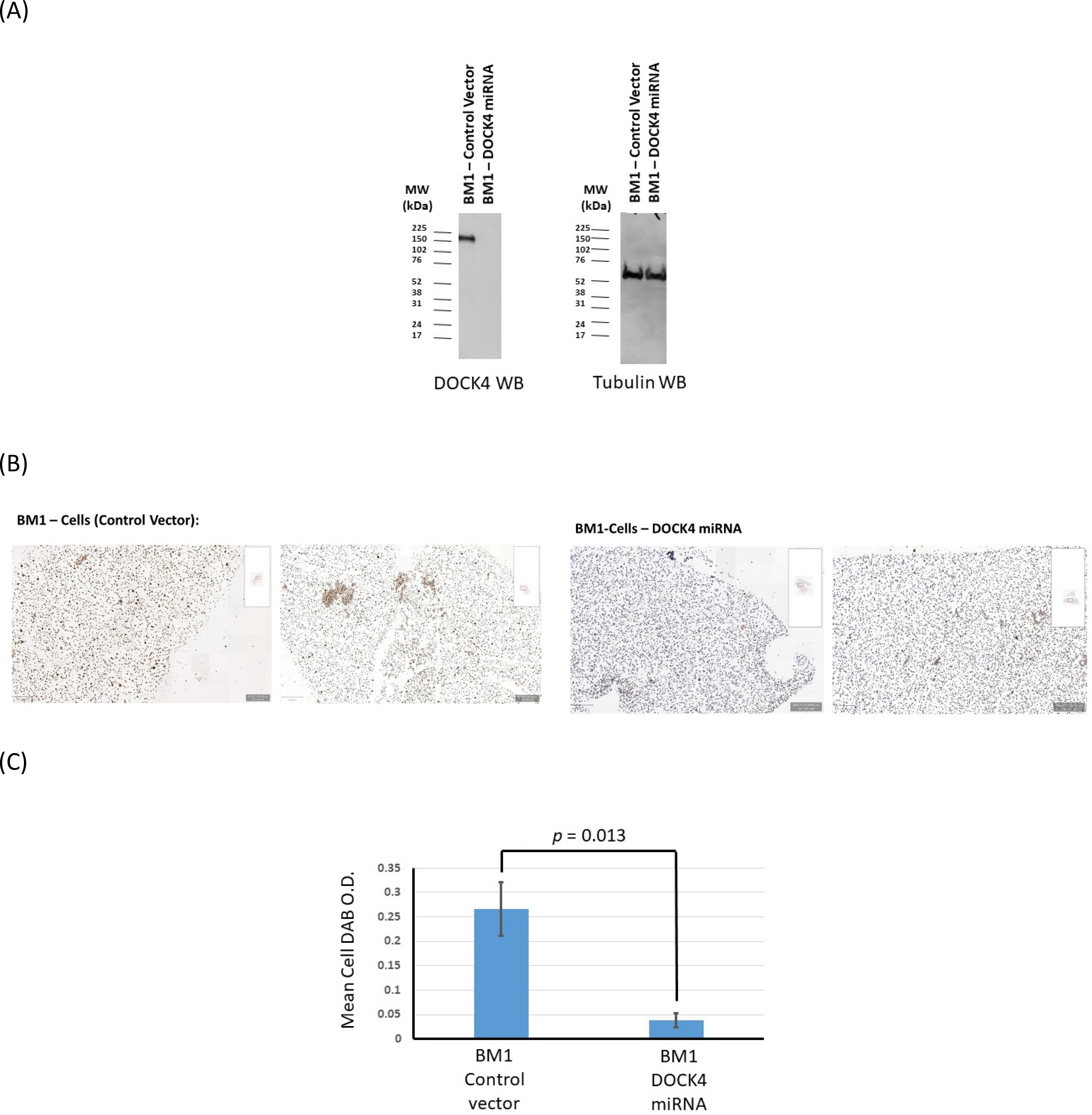


**Figure S2. Testing antibody specificity by immunostaining of FFPE cells.**
(A) Western blotting confirmation of DOCK4 knockdown in DOCK4 miRNA transfected MDA-MB-231 cells. (B) Representative microscopy fields from FFPE bone-homing MDA-MB-231 cells (BM1) stably transfected with either control lentivirus (control vector) or DOCK4-miRNA-expressing lentivirus (DOCK4-miRNA) and subjected to immunostaining using the Bethyl anti-DOCK4 antibody and a DAB colour reaction with haematoxylin counterstaining. Two representative fields are shown for each cell type. (C) Quantification of mean cellular DAB staining level across five replicates of each cell type (mean ± SEM, *n* = 5).

(A)

**
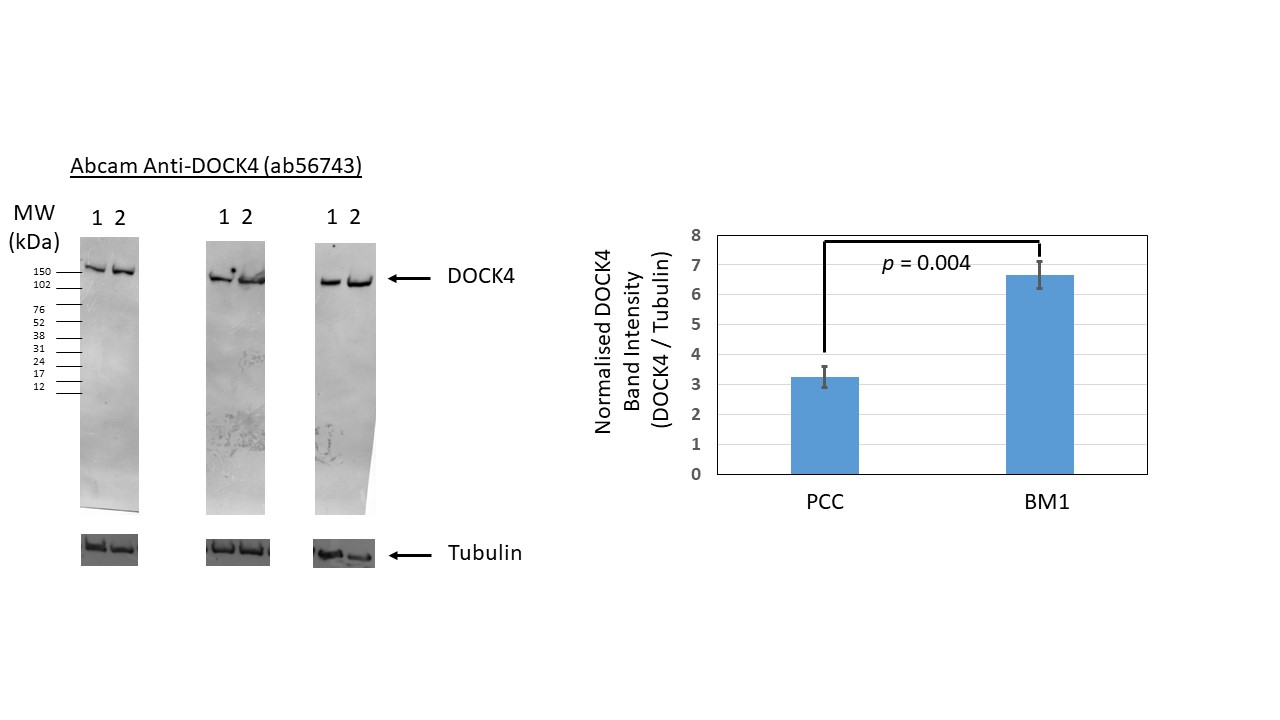
**

(B)

**
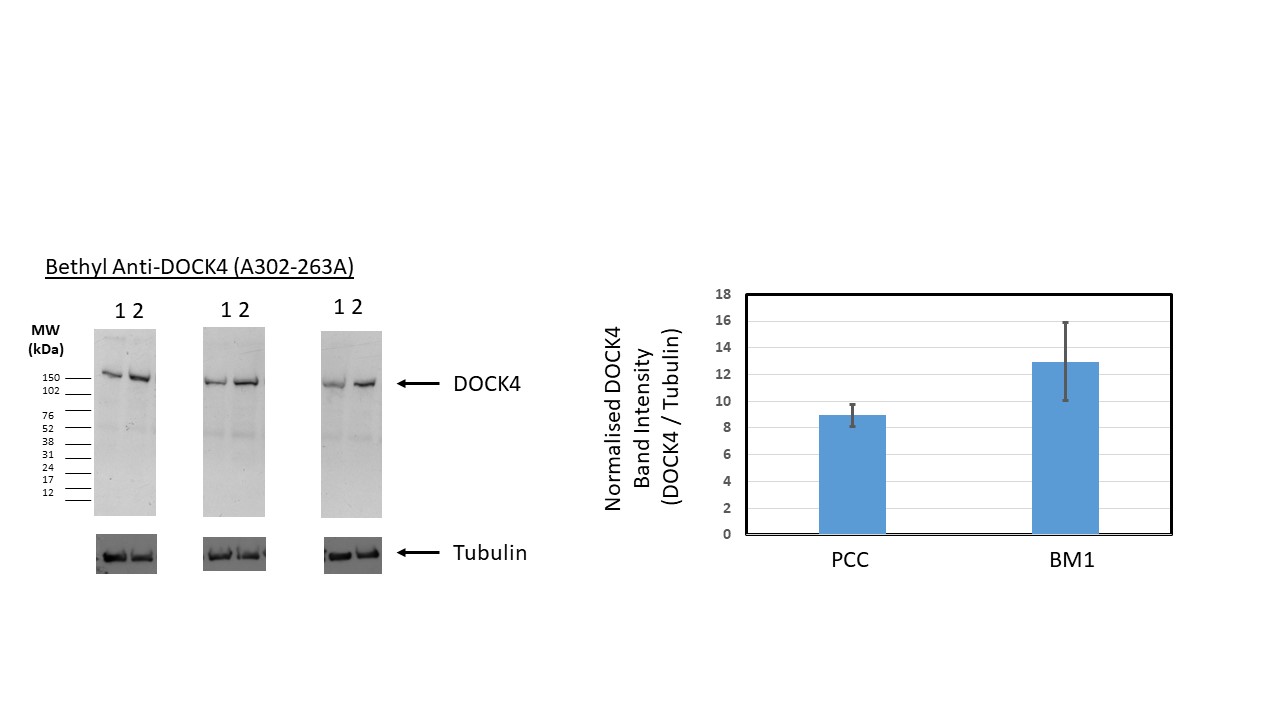
**

**Figure S3. Testing antibody specificity by western blotting of cell lysates.**(A) Full gel length ECL images of western blots of 50 µg total cell lysate from PCC cells
(lane 1) and BM1 cells (lane 2). Western blotting was performed using the Abcam anti-DOCK4 antibody (ab56743) as described. A tubulin loading control is also shown. Quantification of the normalised DOCK4 band intensity is depicted in the attached histogram (*n* = 3 replicate gels, mean band intensity ± SEM). (B) Full gel length ECL images of western blots of 50 µg total cell lysate from PCC (lane 1) and BM1 cells (lane 2) probed with the Bethyl anti-DOCK4 antibody. A tubulin loading control was included. Histogram depicts the quantification of the normalised DOCK4 band intensity (mean ± SEM, *n* = 3, replicate gels).

**
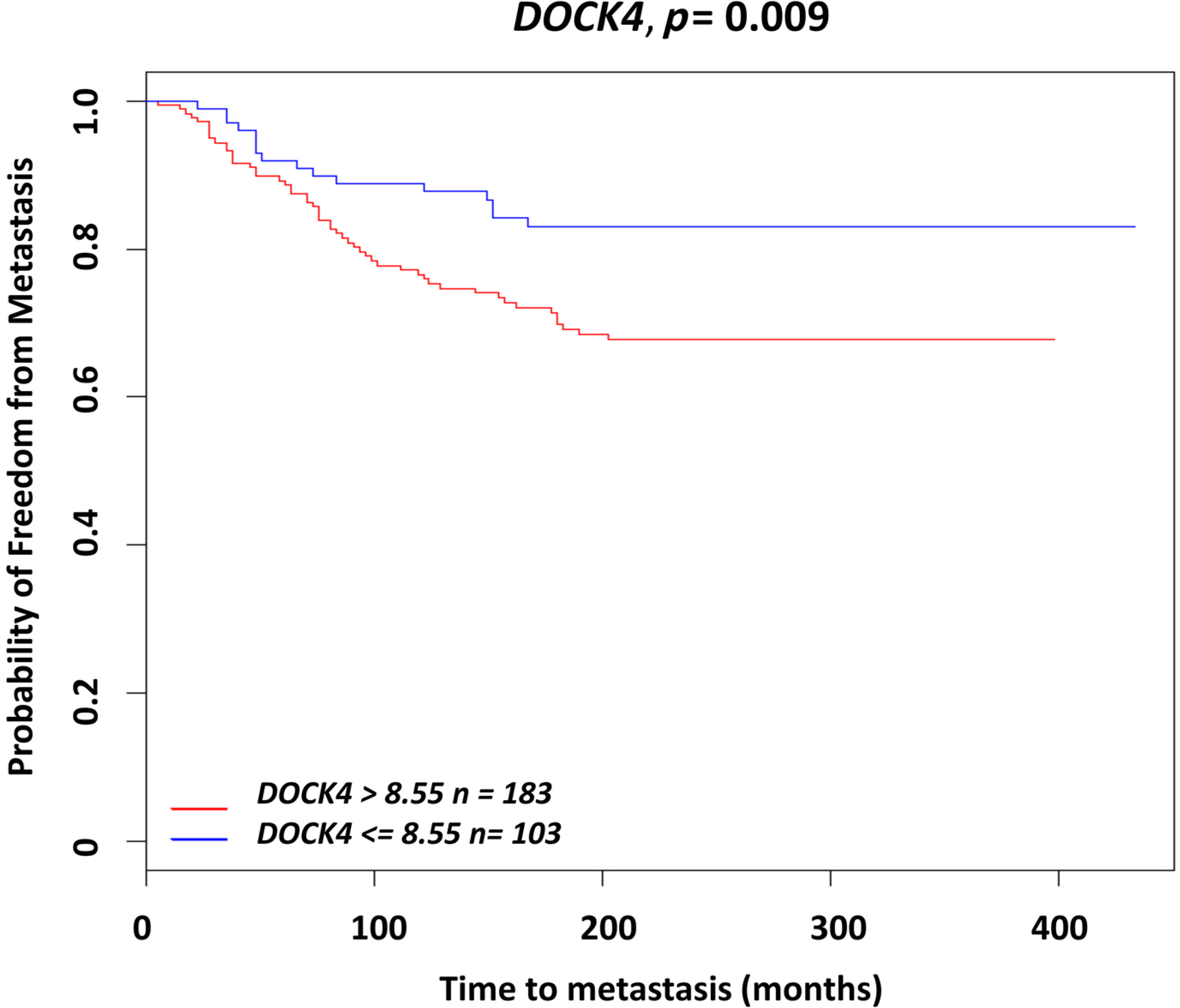
**

**Figure S4. Gene expression analysis of *DOCK4* expression and time to bone metastasis.**Time to bone metastasis analysis of *DOCK4* expression level within breast cancer patients from Wang *et al* [32] with high (>8.53) and low (<8.53) levels of *DOCK4* expression.


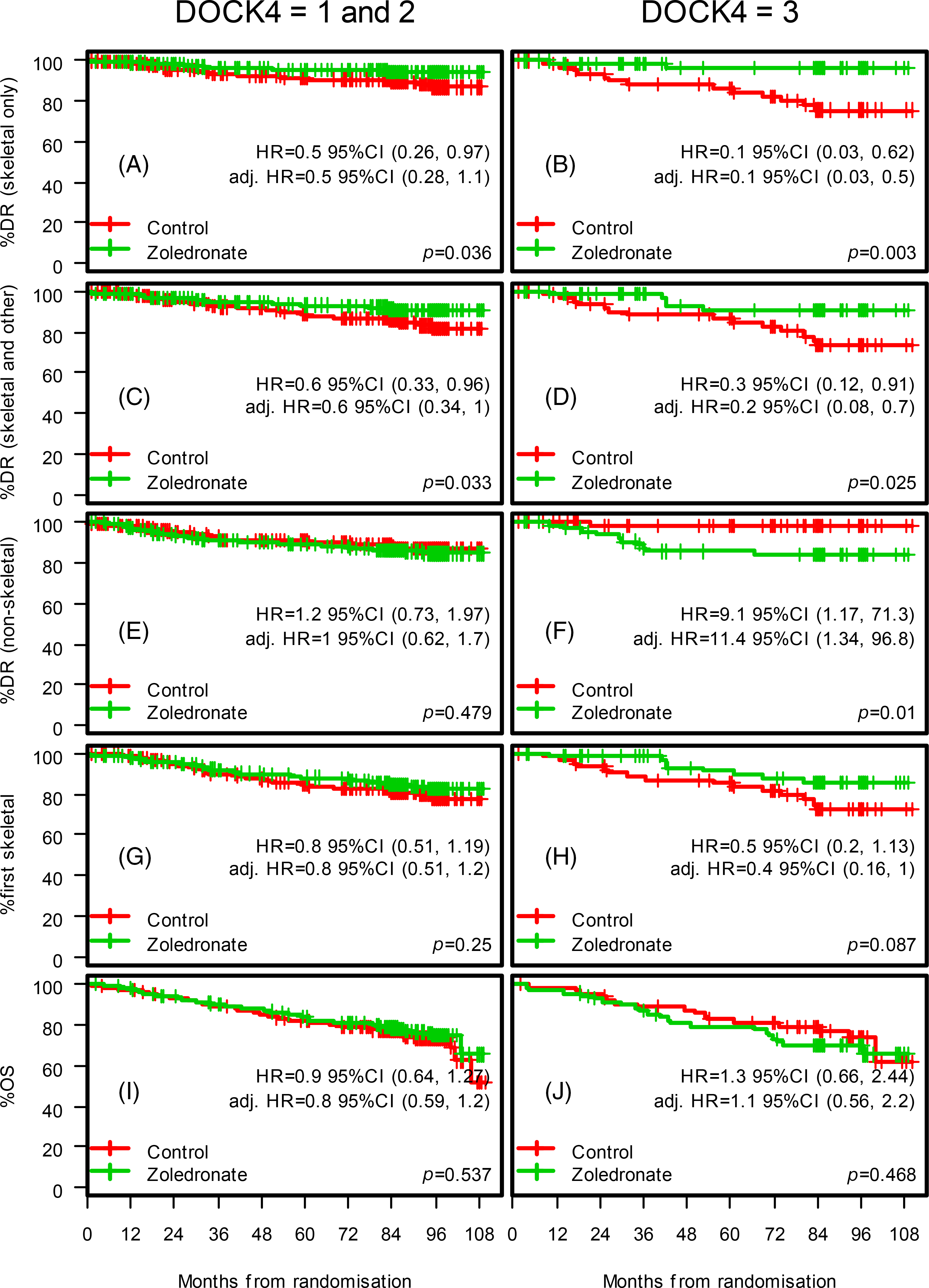


**Figure S5. Univariate associations of distant recurrence outcomes with biomarker expression for DOCK4 low and DOCK4 high.** Estimates are from Cox proportional hazards regressions. Kaplan–Meier estimates of the survival function for time to distant recurrence (DR) and overall survival for control and zoledronate arms for dichotomised DOCK4 low (1 and 2) and high (3). Numbers 1–3 refer to the DOCK4 staining intensity scores. Comparisons shown to be significant are also significant in analyses adjusting for the effect of systemic therapy plan, ER status and lymph node involvement. (A and B) Skeletal only, (C and D) skeletal and other, (E and F) non-skeletal, (G and H), first skeletal irrespective of whether other distant events have occurred previously (i.e. bone metastasis-free survival); (I and J) overall survival. *P* values refer to the log-rank test. For definitions of non-skeletal, skeletal and other and skeletal only see legend to Table 1.
