# Supplementary material for: Identification and validation of DOCK4 as a potential biomarker for risk of bone metastasis development in patients with early breast cancer
Source: J Pathol. 2019 Jan 25;247(3):381–91. doi: 10.1002/path.5197 (PMC6618075; doi:10.1002/path.5197)
Supplement: Supplementary file 3 — Table S1. Proteomic results for up‐regulated proteins within the SILAC comparison of parental (PCC) and bone‐homing (BM1) MDA‐MB‐231 cells. [file PATH-247-381-s003.docx]

**Identification and validation of DOCK4 as a potential biomarker for risk of bone metastasis development in patients with early breast cancer**

Westbrook JA *et al*. *J Pathol* 2018 (DOI: 10.1002/path. 5197)

**Supplementary Table S1.** Proteomic results for upregulated proteins within the SILAC comparison of parental (PCC) and bone-homing (BM1) MDA-MB-231 cells

| **Protein name** | **Gene symbol** | **UniProt ID** | **BM1/PCC Fold-change** | **No. of peptides** | **Mol. weight [kDa]** | **Sequence length** |
| --- | --- | --- | --- | --- | --- | --- |
| Interstitial collagenase | MMP1 | P03956 | 22.56 | 12 | 54.006 | 469 |
| Sperm protein associated with the nucleus on the X chromosome B1 | SPANXB1 | Q9NS25 | 15.58 | 6 | 11.826 | 103 |
| Plasminogen activator inhibitor 2 | SERPINB2 | P05120 | 15.56 | 12 | 46.596 | 415 |
| Protein EVI2B | EVI2B | P34910 | 7.43 | 4 | 50.583 | 463 |
| Creatine kinase B-type | CKB | P12277 | 6.92 | 6 | 42.644 | 381 |
| Regulator of G-protein signaling 10 | RGS10 | O43665 | 4.70 | 7 | 21.21 | 181 |
| Neutral cholesterol ester hydrolase 1 | NCEH1 | Q6PIU2 | 4.69 | 9 | 49.881 | 448 |
| Protein-glutamine gamma-glutamyltransferase 2 | TGM2 | P21980 | 4.40 | 24 | 77.328 | 687 |
| Annexin A3 | ANXA3 | P12429 | 4.08 | 14 | 36.417 | 323 |
| Cell division cycle protein 20 homolog | CDC20 | Q12834 | 3.67 | 4 | 54.722 | 499 |
| CD44 antigen | CD44 | P16070 | 3.22 | 10 | 81.553 | 742 |
| Formin-like protein 1 | FMNL1 | O95466 | 3.19 | 14 | 122.41 | 1104 |
| Alpha-taxilin | TXLNA | P40222 | 2.76 | 6 | 61.89 | 546 |
| Dedicator of cytokinesis protein 4 | DOCK4 | Q8N1I0 | 2.69 | 6 | 225.15 | 1966 |
| Niemann-Pick C1 protein | NPC1 | O15118 | 2.68 | 9 | 142.17 | 1278 |
| Membrane-associated progesterone receptor component 2 | PGRMC2 | O15173 | 2.53 | 7 | 26.17 | 247 |
| Pericentrin | PCNT | O95613 | 2.52 | 6 | 378.08 | 3336 |
| Four and a half LIM domains protein 2 | FHL2 | Q14192 | 2.42 | 3 | 44.205 | 389 |
| Ferritin light chain | FTL | P02792 | 2.38 | 6 | 20.019 | 175 |
| Vimentin | VIM | P08670 | 2.37 | 46 | 53.651 | 466 |
| Serine/threonine-protein phosphatase CPPED1 | CPPED1 | Q9BRF8 | 2.36 | 4 | 35.576 | 314 |
| CUB domain-containing protein 1 | CDCP1 | Q9H5V8 | 2.33 | 4 | 92.901 | 836 |
| Tyrosine-protein phosphatase non-receptor type 1 | PTPN1 | P18031 | 2.31 | 12 | 49.966 | 435 |
| Nicotinamide phosphoribosyltransferase | NAMPT | P43490 | 2.29 | 26 | 55.52 | 491 |
| Xaa-Pro aminopeptidase 1 | XPNPEP1 | Q9NQW7 | 2.27 | 8 | 74.797 | 666 |
| Integrin alpha-6 | ITGA6 | P23229 | 2.25 | 22 | 126.63 | 1130 |
| Cytosolic non-specific dipeptidase | CNDP2 | Q96KP4 | 2.21 | 20 | 52.878 | 475 |
| Fascin | FSCN1 | Q16658 | 2.21 | 21 | 55.135 | 500 |
| Enoyl-CoA delta isomerase 2, mitochondrial | ECI2 | O75521 | 2.16 | 2 | 43.585 | 394 |
| Coiled-coil domain-containing protein 124 | CCDC124 | Q96CT7 | 2.11 | 6 | 25.835 | 223 |
| Glutathione S-transferase Mu 1 | GSTM1 | P09488 | 2.04 | 3 | 25.712 | 218 |
| Cap-specific mRNA | CMTR1 | Q8N1G2 | 2.01 | 5 | 95.32 | 835 |
| Nucleobindin-1 | NUCB1 | Q02818 | 2.00 | 5 | 53.879 | 461 |
| Adenosine kinase | ADK | P55263 | 1.97 | 13 | 40.545 | 362 |
| Unconventional myosin-IXb | MYO9B | Q13459 | 1.96 | 13 | 243.56 | 2158 |
| Stathmin | STMN1 | P16949 | 1.96 | 7 | 17.302 | 149 |
| Non-syndromic hearing impairment protein 5 | DFNA5 | O60443 | 1.96 | 6 | 54.554 | 496 |
| C-1-tetrahydrofolate synthase, cytoplasmic | MTHFD1 | P11586 | 1.92 | 49 | 101.56 | 935 |
| Tryptophan--tRNA ligase, cytoplasmic | WARS | P23381 | 1.92 | 15 | 53.165 | 471 |
| Protein CutA | CUTA | O60888 | 1.90 | 2 | 20.925 | 198 |
| Epidermal growth factor receptor substrate 15-like 1 | EPS15L1 | Q9UBC2 | 1.89 | 9 | 99.606 | 910 |
| Cdc42-interacting protein 4 | TRIP10 | Q15642 | 1.88 | 8 | 68.351 | 601 |
| Mitochondrial import inner membrane translocase subunit Tim9 | TIMM9 | Q9Y5J7 | 1.88 | 3 | 10.378 | 89 |
| Neutral amino acid transporter B | SLC1A5 | Q15758 | 1.81 | 12 | 56.598 | 541 |
| Chloride intracellular channel protein 4 | CLIC4 | Q9Y696 | 1.80 | 17 | 28.772 | 253 |
| Copine-1 | CPNE1 | B0QZ18 | 1.79 | 12 | 59.717 | 542 |
| E3 ubiquitin-protein ligase Itchy homolog | ITCH | Q96J02 | 1.76 | 4 | 102.8 | 903 |
| Nucleosome assembly protein 1-like 1 | NAP1L1 | P55209 | 1.75 | 14 | 45.374 | 391 |
